# To the Editors of the Medical and Physical Journal

**Published:** 1805-01-01

**Authors:** John Whitlam

**Affiliations:** Nottingham


### *To the Editors of the Medical and Physical Journal.*

GENTLEMEN,

IN my communication on the Management of Leeches, I purposely avoided hypothetical speculations; well knowing that they frequently mislead, instead of instructing us.

There have been two methods strongly recommended for the preservation of these useful little animals; but *experience alone* will determine, which we ought to prefer. The same unerring guide would, perhaps, lead us to make farther discoveries respecting them, were we to imitate their native ponds, by acquiring an exact knowledge of the contents of the water, &c. in which they are most plentifully produced.

We might then, by exposing them at different periods to the action of the atmosphere, when it undergoes its greatest variety of changes, observe their resources when they attempt to avoid the inconveniences of a change of temperature, or the sudden afflux of a fresh quantity of the surrounding medium.

As experiments of this kind, which are at once both useful and amusing, may be made without much trouble or expence, I should hope that some of your Correspondents will apply themselves, and give the result of their observations through the medium of your valuable publication. I am, &c.

*Nottingham, Dec.* 5, 1805. JOHN WHITLAM.

---

